# Validation of a food-frequency questionnaire for assessing vitamin intake of Japanese women in early and late pregnancy with and without nausea and vomiting

**DOI:** 10.1017/jns.2016.14

**Published:** 2016-07-07

**Authors:** Seung Chik Jwa, Kohei Ogawa, Minatsu Kobayashi, Naho Morisaki, Haruhiko Sago, Takeo Fujiwara

**Affiliations:** 1 Department of Social Medicine, National Research Institute for Child Health and Development, National Center for Child Health and Development, 2-10-1 Okura, Setagaya-ku, Tokyo 157-8535, Japan; 2 Center of Maternal–Fetal, Neonatal and Reproductive Medicine, National Center for Child Health and Development, 2-10-1 Okura, Setagaya-ku, Tokyo 157-8535, Japan; 3 Department of Food Science, Otsuma Women's University, 12 Sanban-cho, Chiyoda-ku, Tokyo 102-8357, Japan; 4 Department of Global Health Promotion, Tokyo Medical and Dental University, 1-5-45, Yushima, Bunkyo-ku, Tokyo 113-8519, Japan

**Keywords:** Pregnancy, Food-frequency questionnaires, Vitamins, Validation, Nausea, Vomiting, 25(OH)D, 25-hydroxyvitamin D, FFQ1, first FFQ between 5 and 15 weeks of gestation, FFQ2, second FFQ between 22 and 35 weeks of gestation, NCCHD, National Center for Child Health and Development, NVP, nausea and vomiting during pregnancy, NVP (+), participants who reported their dietary intake was reduced by NVP, NVP (–), participants whose food consumption had not decreased

## Abstract

Maternal vitamin intake during pregnancy is crucial for pregnancy outcomes and the child's subsequent health. However, there are few valid instruments for assessing vitamin intake that address the effects of nausea and vomiting during pregnancy (NVP). This study aimed to investigate the validity of a FFQ concerning vitamin intake during early and late pregnancy with and without NVP. The participants comprised 200 Japanese pregnant women who completed the FFQ and from whom blood samples were taken in early and late pregnancy. Energy-adjusted dietary vitamin intakes (vitamin C, folate, vitamin B_6_, vitamin B_12_, vitamin A, vitamin E and vitamin D) from FFQ were compared with their blood concentrations. A subgroup of women with NVP was investigated. In early pregnancy, significant correlations between FFQ and biomarkers were observed for vitamin C (*r* 0·27), folate (*r* 0·18) and vitamin D (*r* 0·26) in women with NVP and for vitamin A (*r* 0·18), vitamin B_12_ (*r* 0·24) and vitamin D (*r* 0·23) in women without NVP. No significant correlations were observed in either group for vitamins B_6_ or E. In late pregnancy, similar significant associations were observed for vitamin C (*r* 0·27), folate (*r* 0·22), vitamin B_6_ (*r* 0·18), vitamin B_12_ (*r* 0·27) and vitamin A (*r* 0·15); coefficients were higher among women without NVP. Our study demonstrates that the FFQ is a useful tool for assessing intake of several important vitamins in early and late pregnancy regardless of NVP status.

A balanced vitamin intake during pregnancy is crucial not only for maternal and fetal health, but also for the subsequent health of the child^(^^1^^,^^2^^)^. Maternal folic acid supplements from before conception through early pregnancy reportedly decrease the risk of fetal neural tube defects^(^^3^^)^, whereas excess vitamin A intake is associated with increased risk of central neural crest defects^(^^4^^)^. Low maternal plasma concentrations of 25-hydroxyvitamin D (25(OH)D) during pregnancy are associated with pre-eclampsia^(^^5^^)^, gestational diabetes mellitus^(^^6^^)^ and Caesarean section in mothers, and various other complications for the child, such as preterm delivery^(^^7^^–^^9^^)^, allergic diseases and depression^(^^10^^–^^12^^)^. Maternal nutrition during pregnancy is an increasingly important topic, prompting a demand for the development of valid instruments for assessing maternal consumption of food and nutrients, including use of supplements, during pregnancy.

Several tools are currently available for assessing nutritional intakes, including of vitamins. While food records and 24 h dietary recalls may provide an accurate estimate on diet, it is not economically feasible to administer them in epidemiological studies. Furthermore, although food records are usually considered the ‘gold standard’ measurement, they require a high degree of participant cooperation and literacy. Similarly determination of serum biomarker concentrations, which are also ‘gold standard’ measurements, are expensive and invasive.

A FFQ, on the other hand, is a useful low-cost instrument for assessing habitual diet that is easy to implement in large epidemiological studies. FFQ have been validated in various populations^(^^13^^)^. However, FFQ regarding dietary changes and supplement use during pregnancy have less frequently been validated^(^^14^^)^.

Maternal diets change over the course of a pregnancy^(^^15^^,^^16^^)^ and dietary intake can be significantly altered by nausea and vomiting during pregnancy (NVP)^(^^17^^,^^18^^)^. No previous studies have investigated the effects of NVP on the validity of a FFQ during pregnancy. Although many epidemiological studies have used biomarker concentrations in mid and late pregnancy as indicators of maternal nutritional status^(^^6^^,^^19^^–^^21^^)^, those values can be affected by plasma volume increases^(^^22^^,^^23^^)^. Hence, other means of investigating the associations between oral nutritional intakes and biomarkers during different gestational periods are required.

Here, we investigated the validity of a FFQ concerning vitamin intake during early and late pregnancy among Japanese women, including assessing the impact of NVP on dietary intake and validity.

## Materials and methods

### Study design and subjects

A FFQ for Japanese women in early and late pregnancy was investigated by comparing data derived from it with blood biomarker concentrations. The study was conducted at the National Center for Child Health and Development (NCCHD, Tokyo, Japan). Between May 2011 and February 2012, participants were randomly recruited from the out-patient department of obstetrics during their first prenatal visit from 5 to 15 weeks of gestation. Of the 272 women asked to participate in our study, 248 (91·2 %) eventually consented to enrolment. Among those 248 women, twenty-one (8·5 %) withdrew after initially participating and another twenty-seven were excluded from the analysis because of severe emesis (*n* 3) or unavailable FFQ (*n* 21) or biomarker data (*n* 3) in early pregnancy. Thus, the final analysis included 200 participants, 154 of whom completed both the FFQ and a blood test in late pregnancy. For all participants, the estimated due date for delivery was confirmed by ultrasonography at recruitment. Written informed consent for participation was obtained from all participants at recruitment. The Institutional Review Board of the NCCHD approved this study (Institutional Review Board approval no. 461).

### Assessment of nausea and vomiting during pregnancy

Information on NVP was collected by a questionnaire on enrolment that offered the following seven options in answer to a question about how much participants’ dietary intakes were influenced by NVP: (1) dietary intake increased after pregnancy; (2) dietary intake did not change and I had no symptoms of NVP; (3) dietary intake did not change but I felt nauseous; (4) dietary intake decreased by 10–50 % compared with before conception because of NVP; (5) dietary intake decreased by 50–80 % compared with before conception because of NVP; (6) dietary intake decreased more than 80 % compared with before conception because of NVP; and (7) I could not eat at all because of NVP (i.e. severe emesis). Three participants who answered with option 7 were excluded from the analysis.

These seven options were used to divide participants into two groups by NVP status. Participants who reported that their dietary intake was reduced by NVP were classified as the NVP (+) group and those whose food consumption had not decreased were classified as NVP (−). The validity of the question for NVP was investigated by comparing change in body weight (kg) from before pregnancy to at the time of administration of the FFQ1. The same questionnaire was also used to assess NVP status in late pregnancy, together with a FFQ2.

### FFQ

Participants were asked to complete the FFQ twice during their pregnancies: the first FFQ between 5 and 15 weeks of gestation (FFQ1); and the second between 22 and 35 weeks of gestation (FFQ2). Both FFQ asked for average food consumption in the 2 months prior to completing the FFQ. Each FFQ consisted of 167 food and beverage items and nine frequency categories. For the latter, the rankings ranged from ‘almost never’ to ‘seven or more times per d’ (or ‘10 glasses per d’ for beverages). The food items in the current study were based on the food list in the Japan Public Health Center-based Prospective Study^(^^24^^)^ with the following modifications. Because our study was conducted in an urban community, six food items were added to the list: ground meat; pastry; cornflakes; pudding; jelly; and cocktail. The following portion sizes were specified for each food item: medium (the standard amount); small (50 % smaller); and large (50 % larger). Intake of energy, thirty-six nutrients, and twenty-six food groups were calculated using a food composition table developed for the FFQ based on the Standardised Tables of Food Composition in Japan (2010 edition).

In the FFQ, participants were asked to provide brand names of supplements and frequency of use ranging from once or twice per week to four or more times per d. Daily vitamin intake from supplements calculated from the supplements’ nutritional compositions were added to the daily nutrient intake from the FFQ to calculate the total daily nutrient intake.

### Biomarkers

Non-fasting blood samples were obtained from each participant on enrolment and in late pregnancy (between 22 and 29 weeks of gestation). They were separated by centrifugation for 5 min at 3000 rpm immediately after venepuncture and stored at −40°C in a laboratory in the NCCHD until being shipped to an external laboratory (SRL, Hachioji, Tokyo, Japan). For serum vitamin E, retinol and vitamin B_6_, blood samples were immediately put into a light-shielded spit after centrifugation to prevent degeneration caused by exposure to light. For vitamin C, 500 µl of each sample were mixed with perchloric acid after centrifugation to exclude protein, further centrifuged at 3000 rpm for 5 min and then put into a light-shielded spit to prevent degeneration. After being packed with dried ice, the samples were carefully transported by the company's daily transportation system to the laboratory for analysis.

Serum vitamin C concentrations were measured by HPLC as described previously^(^^25^^)^ using an HPLC system (Shimadzu) with a commercially available column (Wakosil-II 5C18RS; Wako). Serum folate and vitamin B_12_ were measured by a chemiluminescence enzyme immunoassay using a commercially available kit for folate (Access folate (FOLW); Beckman Coulter) and vitamin B_12_ (Access B_12_; Beckman Coulter). Vitamin B_6_ was assayed by HPLC as pyridoxal using a commercially available column (Wakosil-II 5C18HG, Wako). Serum retinol was also measured by an HPLC method using an HPLC system with a commercially available column (Inertsil ODS; GL Sciences). Serum vitamin E was measured by a fluorescence method with an F-2500 fluorescence spectrophotometer (Hitachi). Serum 25(OH)D in early pregnancy was measured by radioimmunoassay using a commercially available kit (25-HydroxyvitaminD 125I RIA Kit; DiaSorin). For logistic reasons, the serum 25(OH)D concentrations of 110 participants in mid-pregnancy were measured by a different external laboratory (LSI Medience, Itabashi, Tokyo, Japan) by a competitive protein-binding assay as previously reported^(^^26^^)^.

The intra-assay CV were as follows: vitamin C, 2·4–7·9 %; folate, 3·0–6·5 %; vitamin B_6_, 3·7–9·2 %; vitamin B_12_, 5·7–8·0 %; retinol, 8·7–13·2 %; vitamin E, 1·0–6·4 %; and 25(OH)D, 3·0–9·5 % in early pregnancy and 7·7–10·9 % in late pregnancy. The corresponding inter-assay CVs were as follows: vitamin C, 0·0–4·4 %; folate, 2·4–3·8 %; vitamin B_6_, 0·0–2·6 %; vitamin B_12_, 3·6–6·0 %; retinol, 0·0 %; vitamin E, 1·5–4·2 %; and 25(OH)D, 5·9–9·5 % in early pregnancy and 8·6–11·6 % in late pregnancy.

### Demographic information

Participants’ ages, parity and socio-economic status were collected by a self-administered questionnaire on enrolment. Body weight pre-pregnancy and at FFQ1 and height were self-reported. Pre-pregnancy BMI was calculated as pre-pregnancy body weight (kg)/height^2^ (m) and categorised according to the criteria of the WHO as underweight (BMI < 18·5 kg/m^2^), normal weight (BMI 18·5–25 kg/m^2^) and overweight or obese (BMI > 25·0 kg/m^2^).

### Statistical analysis

Baseline characteristics were compared according to NVP status in early pregnancy. Mean vitamin intakes were calculated from the FFQ1 and FFQ2. For biomarkers and FFQ, differences between NVP statuses were investigated by Student's *t* test. Paired *t* tests were also used to assess differences in nutritional intake or serum biomarker concentrations between early and late pregnancy. All vitamin intakes calculated from FFQ were log-transformed to improve normality. Energy-adjusted intakes were calculated by the residual method^(^^27^^)^. Spearman correlation coefficients were calculated to assess the validity of the FFQ by comparing crude or energy-adjusted intakes derived from the FFQ1 and FFQ2 with biomarkers measured at the corresponding period. Because serum folate concentrations of more than 20 ng/ml were coded as ‘>20 ng/ml’, those values were imputed as 21 ng/ml for the purposes of analysis (seventeen participants in early pregnancy) with the aims of maintaining sample numbers and avoiding overestimation. Spearman correlation coefficients by NVP status were separately calculated for early and late pregnancy. Correlation coefficients were also calculated, including for intake from supplement use. Furthermore, all pregnant women were classified into quintiles according to their energy-adjusted vitamin intakes and serum concentrations. Agreement in quintile orders were assessed as the percentage classification for participants based on energy-adjusted dietary intakes and serum concentrations in the same and adjacent quintiles. All analyses were performed with the STATA/SE statistical package, version 12.1 (StataCorp LP). Two-tailed *P* values <0·05 were considered as statistically significant.

## Results

The participants’ characteristics are shown in Table 1. There were 108 (54 %) women with NVP in early pregnancy. Mean pre-pregnancy body weight was 50·5 (sd = 6·2) kg in the NVP (+) group, whereas that in the NVP (−) group was 51·6 (sd = 6·7) kg; this difference is not statistically significant (*P* = 0·23). Mean body-weight change from pre-pregnancy to the time of completing the FFQ1 was −0·28 (sd = 2·0) kg in the NVP (+) group and +0·90 (sd = 2·1) kg in the NVP (−) group, this difference being significant (*P* = 0·0001). There was a significant linear trend between NVP assessment scale and body-weight change (kg) (coefficient (kg) = −0·44, 95 % CI −0·63, 0·25; *P* < 0·001). Mean maternal age was 35·2 (sd = 4·1) years; 66 % of participants were nulliparous, 64·5 % had graduated from college or university, and 43·5 % were classified as having annual household incomes of more than JPY10 million (equivalent to US$ 83 000). More than 22 % of participants’ pre-pregnancy BMI values were less than 18·5 kg/m^2^, whereas 4 % were more than 25 kg/m^2^. Overall, 8·5 % of participants took a folic acid supplement more than once per d. Women with NVP were more likely to have the lowest educational level (23·2 %) than those without NVP (12·0 %) and pre-pregnancy BMI <18·5 kg/m^2^ (25·0 % in the NVP *v*. 19·6 % in the non-NVP group).

**Table 1.** Characteristics of the study participants (*n* 200) (Number of subjects and percentages, mean values and standard deviations)

	Total (*n* 200)		NVP (+) (*n* 108)		NVP (−) (*n* 92)	
Characteristic	*n*	%	*n*	%	*n*	%
Gestational weeks at FFQ1 (weeks)						
Mean	10·2		10·5		9·9	
sd	1·5		1·4		1·5	
Gestational weeks at FFQ2 (weeks)						
Mean	29·1		29·2		29·0	
sd	1·8		1·7		1·8	
Maternal age (years)	35·2	4·1	34·7	4·2	35·7	3·8
≤29 years	22	11·0	16	14·8	6	6·5
30–34 years	60	30·0	33	30·6	27	29·4
35–39 years	92	46·0	46	42·6	46	50·0
≥40 years	26	13·0	13	12·0	13	14·1
Parity						
0	132	66·0	72	66·7	60	65·2
≥1	68	34·0	36	33·3	32	34·8
Educational level						
Junior high school, high school or vocational training school	36	18·0	25	23·2	11	12·0
Some college	35	17·5	14	13·0	21	22·8
College or more	129	64·5	69	63·9	60	65·2
Annual household income						
<4 million yen	15	7·5	8	7·4	7	7·6
4–6 million yen	35	17·5	21	19·4	14	15·2
6–8 million yen	27	13·5	10	9·3	17	18·5
8–10 million yen	33	16·5	20	18·5	13	14·1
Over 10 million yen	87	43·5	47	43·5	40	43·5
Missing	3	1·5	2	1·9	1	1·1
Pre-pregnancy body weight (kg)						
Mean	51·0		50·5		51·6	
sd	6·4		6·2		6·7	
Body weight at FFQ1 (kg)						
Mean	51·3		50·2		52·5	
sd	6·8		6·5		6·9	
Pre-pregnancy BMI						
<18·5 kg/m^2^	45	22·5	27	25·0	18	19·6
18·5–25 kg/m^2^	146	73·0	76	70·4	70	76·1
≥25·0 kg/m^2^	8	4·0	5	4·6	3	3·3
Missing	1	0·5	0	0·0	1	1·1
Smoking during pregnancy						
Current	1	0·51	1	0·93	0	0·0
Former	11	5·6	4	3·7	7	7·6
Alcohol intake from FFQ1	46	23·0	25	23·2	21	22·8
Use of a folic acid supplement from FFQ1						
More than 4 times per d	1	0·5	0	0·0	1	1·1
2–3 times per d	2	1·0	0	0·0	2	2·2
Once per d	14	7·0	6	5·6	8	8·7
5–6 times per week	2	1·0	1	0·9	1	1·1
3–4 times per week	1	0·5	0	0·0	1	1·1

NVP, nausea and vomiting during pregnancy; NVP (+), participants who reported their dietary intake was reduced by NVP; NVP (–), participants whose food consumption had not decreased; FFQ1, first FFQ between 5 and 15 weeks of gestation; FFQ2, second FFQ between 22 and 35 weeks of gestation.

Mean vitamin intakes based on FFQ1 and FFQ2 are shown in Table 2. In early pregnancy, the mean intake of folate was significantly lower in women with NVP than in those without it (*P* = 0·04). After including supplement intake, α-tocopherol and vitamin D intakes were lower in women with NVP than in those without it; this difference was with marginal significance (*P* = 0·08). Intakes of vitamins C, B_6_, B_12_ and A appeared higher in women without NVP; however, this difference was not statistically significant. Among the 154 participants who completed the FFQ and blood tests in both early and late pregnancy, seventy-nine (51 %) still had NVP in late pregnancy. In late pregnancy, mean intakes of folate were significantly lower in women with NVP (*P* = 0·008). Similarly, mean intakes of vitamins C, B_6_, A and E were lower in women with NVP than in those without it, this difference being marginally significant. Vitamins B_12_, A and D intakes were significantly higher at the time of the FFQ2 than at the time of the FFQ1 (*P* < 0·05).

**Table 2.** Nutritional characteristics assessed by FFQ1 and FFQ2 (Mean values and standard deviations)

	FFQ1							FFQ2							
	Total (*n* 200)		NVP (+) (*n* 108)		NVP (−) (*n* 92)			Total (*n* 154)		NVP (+) (*n* 79)		NVP (−) (*n* 75)			
Nutritional intake	Mean	sd	Mean	sd	Mean	sd	P*	Mean	sd	Mean	sd	Mean	sd	P*	*P*†
Vitamin C (mg/d)	111	61	109	68	112	51	0·78	108	56	100	46	117	65	0·07	0·78
Diet + supplements	142	152	139	163	146	138	0·74	132	144	121	107	143	175	0·34	0·69
Folate (μg/d)	285	129	268	115	305	141	0·04	285	118	261	100	311	131	0·008	0·80
Diet + supplements	323	187	288	144	365	221	0·003	319	165	285	128	355	191	0·008	0·42
Vitamin B_6_ (mg/d)	1·2	0·46	1·1	0·46	1·2	0·45	0·22	1·2	0·43	1·1	0·40	1·3	0·44	0·07	0·24
Diet + supplements	2·0	5·2	1·9	4·9	2·1	5·5	0·77	1·5	2·1	1·5	2·7	1·5	1·3	0·98	0·34
Vitamin B_12_ (μg/d)	4·3	2·7	4·1	2·5	4·5	3·0	0·27	5·1	3·0	5·0	3·0	5·2	2·9	0·76	0·0007
Diet + supplements	5·1	7·8	5·3	10·1	4·9	3·6	0·75	5·4	3·4	5·2	3·2	5·5	3·4	0·53	0·009
Vitamin A (μg/d)	918	695	860	496	985	871	0·21	1004	561	920	488	1093	620	0·06	0·046
Diet + supplements	926	700	866	496	997	879	0·19	1018	578	929	497	1110	643	0·052	0·04
Vitamin E (mg/d)	7·2	3·4	7·0	3·6	7·4	3·0	0·43	7·4	3·1	6·9	3·1	7·9	3·2	0·053	0·12
Diet + supplements	8·8	13·1	7·3	4·7	10·5	18·6	0·08	7·9	4·9	7·4	4·6	8·6	5·1	0·12	0·73
Vitamin D (μg/d)	4·2	2·8	3·9	2·7	4·6	2·8	0·12	5·2	3·4	4·9	3·4	5·5	3·5	0·31	<0·0001
Diet + supplements	4·4	2·8	4·0	2·8	4·7	2·9	0·08	5·5	3·6	5·1	3·4	5·9	3·8	0·19	<0·0001

FFQ1, first FFQ between 5 and 15 weeks of gestation; FFQ2, second FFQ between 22 and 35 weeks of gestation; NVP, nausea and vomiting during pregnancy; NVP (+), participants who reported their dietary intake was reduced by NVP; NVP (–), participants whose food consumption had not decreased.

* *P* value comparing NVP (+) with NVP (−) by Student's *t* test.

† *P* value comparing FFQ2 with FFQ1 (total) by paired *t* test.

Serum concentrations of vitamins in early and late pregnancy are shown in Table 3. In early pregnancy, mean serum folate and 25(OH)D concentrations were significantly higher in women without NVP than in those with it. Similarly, vitamin C and B_6_ concentrations appeared lower in women without NVP than in those with it, this difference being marginally significant (*P* = 0·06). In late pregnancy, serum retinol concentrations were significantly higher in women without NVP than in those with it, whereas serum vitamin E concentrations were higher in women with NVP. Compared with those in early pregnancy, serum concentrations of vitamin C, folate, vitamin B_12_ and vitamin A were significantly lower in late pregnancy; a significant increase was observed in vitamin E concentrations.

**Table 3.** Nutritional characteristics assessed on blood samples obtained in early and late pregnancy (Mean values and standard deviations)

	Blood sample at early pregnancy							Blood sample at late pregnancy							
	Total (*n* 200)		NVP (+) (*n* 108)		NVP (−) (*n* 92)			Total (*n* 154)		NVP (+) (*n* 79)		NVP (−) (*n* 75)			
Serum concentration	Mean	sd	Mean	sd	Mean	sd	P*	Mean	sd	Mean	sd	Mean	sd	P*	*P*†
Vitamin C (μg/ml)	8·9	2·8	8·5	2·5	9·3	3·0	0·06	7·9	2·2	7·6	2·1	8·2	2·2	0·10	0·0001
Folate (ng/ml)	11·6	5·0	10·9	5·0	12·5	5·0	0·03	8·3	4·2	8·0	4·3	8·7	4·2	0·32	<0·0001
Vitamin B_6_ (ng/ml)	14·6	18·2	12·4	13·2	17·3	22·5	0·06	13·2	53·4	13·0	45·4	13·3	61	0·97	0·47
Vitamin B_12_ (pg/ml)	322	126	329	146	313	98	0·37	248	88	243	88	255	88	0·39	<0·0001
Retinol (IU/l)	1100	220	1080	190	1130	240	0·11	1040	210	1010	230	1090	200	0·02	0·0009
Vitamin E (mg/l)	10·0	2·3	10·4	2·2	10·4	2·5	0·93	14·0	2·7	14·8	2·9	13·6	2·4	0·005	<0·0001
25(OH)D (ng/ml)	19·7	7·9	18·4	7·2	21·1	8·6	0·02	20·4‡	6·9	19·9‡	6·9	20·9‡	7·0	0·46	0·55

NVP, nausea and vomiting during pregnancy; NVP (+), participants who reported their dietary intake was reduced by NVP; NVP (–), participants whose food consumption had not decreased.

* *P* value comparing NVP (+) with NVP (−).

† *P* value comparing Blood sample at early pregnancy with Blood sample at late pregnancy by paired *t* test.

‡ *n* 110 (55 for NVP (+) and 55 for NVP (−), respectively).

Spearman correlation coefficients for serum vitamin concentrations in early pregnancy and oral intake assessed from the FFQ1 are shown in Table 4. The correlation coefficients based on crude vitamin intakes indicate a significant correlation for vitamin D (*r* 0·29; *P* < 0·0001), and marginal significance for vitamin B_12_ (*r* 0·13; *P* = 0·06) and vitamin A (*r* 0·13; *P* = 0·08). The correlation coefficients for energy-adjusted vitamin intakes were slightly higher for vitamin C, folate and vitamin B_6_, whereas those of vitamins A, E and D were lower compared with crude intake. The highest energy-adjusted correlation coefficient was for vitamin D (*r* 0·26), followed by vitamin C (*r* 0·14) and vitamin B_12_ (*r* 0·13). When considering vitamin intake from supplements, correlation coefficients improved substantially and significantly for folate (from 0·053 to 0·16), vitamin C (from 0·14 to 0·21) and vitamin B_12_ (from 0·13 to 0·17). Mean percentage agreement was 54·8 %, the highest agreement being observed for vitamin D (60·5 %).

**Table 4.** Spearman correlation coefficients for serum micronutrient concentrations in early gestation and oral intake assessed with FFQ1 stratified by nausea and vomiting during pregnancy (NVP)

	Total (*n* 200)					NVP (+) (*n* 108)					NVP (−) (*n* 92)				
	Crude	*P*	Energy-adjusted	*P*	Adjusted agreement	Crude	*P*	Energy-adjusted	*P*	Adjusted agreement	Crude	*P*	Energy-adjusted	*P*	Adjusted agreement
Vitamin C	0·10	0·14	0·14	0·05	57·0	0·15	0·12	0·2	0·04	50·9	0·015	0·89	0·05	0·65	60·9
Diet + supplements	0·16	0·02	0·21	0·004	58·5	0·20	0·03	0·27	0·006	55·6	0·08	0·44	0·11	0·31	62·0
Folate*	0·024	0·73	0·053	0·46	52·5	0·01	0·89	0·08	0·42	58·3	0·02	0·88	−0·01	0·91	51·1
Diet + supplements	0·14	0·05	0·16	0·03	56·0	0·12	0·21	0·18	0·07	61·1	0·13	0·21	0·10	0·32	62·0
Vitamin B_6_	−0·04	0·53	0·02	0·83	50·0	−0·18	0·06	0·07	0·50	54·6	0·12	0·26	−0·07	0·52	48·9
Diet + supplements	0·02	0·8	0·05	0·51	49·5	−0·13	0·18	0·09	0·37	52·8	0·19	0·08	0·01	0·92	47·8
Vitamin B_12_	0·13	0·06	0·13	0·07	54·0	0·05	0·62	0·07	0·44	56·5	0·24	0·02	0·18	0·09	50·0
Diet + supplements	0·17	0·02	0·17	0·02	57·0	0·07	0·5	0·10	0·29	59·3	0·31	0·003	0·24	0·02	53·3
Vitamin A	0·13	0·08	0·08	0·27	55·0	0·01	0·93	−0·02	0·82	51·9	0·21	0·04	0·19	0·08	60·9
Diet + supplements	0·12	0·096	0·07	0·32	54·0	−0·003	0·97	−0·04	0·71	50·9	0·21	0·04	0·18	0·08	59·8
Vitamin E	0·011	0·88	−0·03	0·71	51·0	0·21	0·03	0·07	0·47	51·9	−0·24	0·02	−0·18	0·09	48·9
Diet + supplements	0·012	0·87	0·05	0·49	52·0	0·21	0·03	0·07	0·46	51·9	−0·11	0·32	−0·15	0·16	52·2
Vitamin D	0·29	<0·0001	0·26	0·0002	60·5	0·30	0·002	0·27	0·005	59·3	0·29	0·03	0·21	0·04	59·8
Diet + supplements	0·3	<0·0001	0·27	0·0001	60·0	0·31	0·001	0·26	0·006	59·3	0·25	0·02	0·23	0·03	60·9

FFQ1, first FFQ between 5 and 15 weeks of gestation; NVP (+), participants who reported their dietary intake was reduced by NVP; NVP (–), participants whose food consumption had not decreased.

* Replaced serum folate level of more than 20 ng/ml with 21 ng/ml (*n* 17).

In women with NVP, significant correlation coefficients were observed for vitamin C (*r* 0·20), vitamin E (*r* 0·21) and vitamin D (*r* 0·31) in crude estimates. Furthermore, folate demonstrated marginal significance (*r* 0·18) in energy-adjusted estimates. Percentage agreement ranged from 50·9 % for vitamin C to 61·1 % for folate. In women without NVP, vitamins B_12_ (*r* 0·31), A (*r* 0·21) and D (*r* 0·29) demonstrated significant correlations for crude estimates whereas vitamin B_6_ demonstrated marginal significance (*r* 0·19). The correlation coefficient for vitamin B_12_ was not significant for energy-adjusted estimates. Percentage agreement ranged from 47·8 % for vitamin B_6_ to 62·0 % for vitamin C.

Spearman correlation coefficients for serum vitamin concentrations in late pregnancy and oral intake assessed with FFQ2 are shown in Table 5. After including vitamin intakes from supplements, significant correlations were observed for folate (*r* 0·15) and vitamin B_12_ (*r* 0·26) in crude estimates. For energy-adjusted intakes, correlation coefficients were significant for vitamin C (*r* 0·27), folate (*r* 0·22), vitamin B_6_ (*r* 0·18), vitamin B_12_ (*r* 0·27), and vitamin A with marginal significance (*r* 0·15). The correlation coefficient for vitamin D was not statistically significant in late gestation. Percentage agreement ranged from 48·7 % for vitamin E to 67·5 % for vitamin C. In women with NVP, energy-adjusted intake showed a significant correlation only for vitamin C (*r* 0·22), whereas energy-adjusted intake in women without NVP showed significant correlations for vitamin C (*r* 0·29), folate (*r* 0·26), vitamin B_6_ (*r* 0·26) and vitamin B_12_ (*r* 0·40).

**Table 5.** Spearman correlation coefficients for serum micronutrient concentrations in late gestation and oral intake assessed with FFQ2 stratified by nausea and vomiting during pregnancy (NVP)

	Total (*n* 154)					NVP (+) (*n* 79)					NVP (−) (*n* 75)				
	Crude	*P*	Energy-adjusted	*P*	Adjusted agreement	Crude	*P*	Energy-adjusted	*P*	Adjusted agreement	Crude	*P*	Energy-adjusted	*P*	Adjusted agreement
Vitamin C	0·12	0·14	0·27	0·0009	67·5	0·13	0·25	0·22	0·047	68·4	0·07	0·55	0·29	0·01	69·3
Diet + supplements	0·07	0·42	0·07	0·42	57·1	0·14	0·23	0·16	0·15	57·0	−0·15	0·19	−0·02	0·88	56·0
Folate	−0·02	0·79	0·05	0·56	55·8	−0·04	0·73	−0·02	0·86	54·4	−0·06	0·60	0·10	0·38	52·0
Diet + supplements	0·15	0·06	0·22	0·008	59·7	0·13	0·25	0·14	0·21	58·2	0·12	0·29	0·26	0·03	57·3
Vitamin B_6_	−0·04	0·62	0·08	0·32	57·8	−0·02	0·85	0·02	0·89	57·0	−0·07	0·53	0·16	0·18	61·3
Diet + supplements	0·06	0·48	0·18	0·03	61·0	0·01	0·91	0·07	0·53	57·0	0·09	0·45	0·26	0·02	61·3
Vitamin B_12_	0·22	0·005	0·25	0·002	60·4	0·16	0·17	0·10	0·36	51·9	0·29	0·01	0·38	0·0008	68·0
Diet + supplements	0·26	0·001	0·27	0·0006	61·0	0·17	0·13	0·13	0·27	54·4	0·33	0·004	0·40	0·0003	68·0
Vitamin A	0·06	0·43	0·16	0·05	59·1	0·02	0·89	0·10	0·39	55·7	0·06	0·63	0·15	0·19	58·7
Diet + supplements	0·06	0·45	0·15	0·07	58·4	0·005	0·97	0·09	0·44	57·0	0·07	0·58	0·15	0·20	57·3
Vitamin E	−0·05	0·58	−0·02	0·79	48·7	0·06	0·61	0·06	0·58	53·2	−0·08	0·51	−0·04	0·75	50·7
Diet + supplements	−0·06	0·45	−0·03	0·74	49·4	0·05	0·65	0·06	0·60	54·4	−0·10	0·38	−0·05	0·65	46·7
Vitamin D*	0·14	0·13	0·09	0·35	54·5	0·11	0·42	0·08	0·55	58·2	0·14	0·31	0·06	0·64	54·5
Diet + supplements	0·14	0·14	0·10	0·29	51·8	0·09	0·51	0·07	0·62	54·5	0·19	0·16	0·11	0·44	52·7

FFQ2, second FFQ between 22 and 35 weeks of gestation; NVP (+), participants who reported their dietary intake was reduced by NVP; NVP (–), participants whose food consumption had not decreased.

* *n* 110.

## Discussion

In our study, more than 50 % of women reported decreased food consumption associated with NVP in both early and late pregnancy. In spite of the influence of NVP on dietary intake during pregnancy, the FFQ accurately estimated intakes of vitamin C, E and D in women with NVP and of vitamins B_12_, A and D in women without NVP in early pregnancy. Moreover, in late pregnancy, the correlations were stronger for many of the vitamins than in early pregnancy, the exceptions being vitamins E and D, for which significant correlations were mainly observed in women without NVP. Thus, we showed that the FFQ can be used to assess vitamin intake in both early and late pregnancy. To our knowledge, this is the first study to demonstrate the validity of an FFQ for assessing the effects of NVP on vitamin intake by measuring their serum concentrations during different gestational periods.

Although correlations between nutritional intakes and biomarkers are reportedly often weaker during pregnancy than in non-pregnancy because of greater intra-individual variability^(^^28^^,^^29^^)^, our results are comparable with those of a previous validation study among pregnant women in which diet history questionnaires were administered to 167 pregnant Japanese women without NVP and not taking supplements, and significant correlations were found for folate (*r* 0·29) and vitamin B_12_ (*r* 0·22)^(^^30^^)^.

Significance of correlation coefficients differed somewhat according to NVP status in our study; in early pregnancy, we identified significant correlations for vitamins C, E and D in women with NVP, whereas we confirmed them for vitamins B_12_, A and D in women without NVP. On the other hand, in late pregnancy we mainly identified significant correlations in women without NVP. This apparent discrepancy could be attributable to dietary changes between the pre-pregnancy period and pregnancy caused by NVP^(^^17^^,^^18^^)^. Based on 3-d food records, women with NVP reportedly have a significantly higher proportion of carbohydrate intake with lower proportions of protein and energy intake^(^^18^^)^. Another study using a FFQ has also demonstrated that, between 18 and 22 weeks of gestation, women with NVP have higher energy intake than women without it, this mainly being attributable to the consumption of sugar-containing soft drinks^(^^17^^)^. In spite of such impacts of NVP on dietary pattern during pregnancy, no previous studies have investigated its effect on the validity of FFQ concerning vitamin intake in early pregnancy^(^^14^^)^. Our study highlights the importance of considering NVP when investigating nutritional intake during pregnancy. Another potential reason for the differences, particularly those we identified in early pregnancy, is that we may have overestimated the nutritional intake of women with NVP because the serum vitamin concentrations of some participants may have been influenced by pre-pregnancy intakes.

Correlation coefficients for vitamin C, folate, and vitamins B_6_, B_12_ and A were higher in late pregnancy than in early pregnancy. This may be due to changes in food choices over the course of the pregnancies^(^^16^^)^. On the other hand, we found poor correlations for vitamin E in both early and late pregnancy, which is comparable with findings of previous studies among pregnant women^(^^31^^,^^32^^)^. It has been suggested that concentrations in other tissues, such as adipose tissue, may more accurately reflect oral vitamin E intake than plasma concentrations of vitamin E^(^^33^^)^.

Interestingly, the correlation coefficient for vitamin D was very high in early pregnancy, but became so low as to be non-significant in late pregnancy. This may be attributable to the effect of gestational period on validity. One previous study demonstrated high correlations between serum 25(OH)D concentrations and FFQ findings in early pregnancy (*r* 0·45)^(^^34^^)^, whereas another study that looked at maternal dietary history and plasma 25(OH)D concentration in late pregnancy reported a poor correlation (*r* 0·07)^(^^35^^)^. It is noteworthy that seasonality may also affect the findings: more than 80 % of the FFQ1 were administered during a relatively warm season in Tokyo when the average monthly temperature was ≥15° (range 18·5–27·5°), whereas most of the FFQ2 were administered in a cooler season when the average monthly temperature was ≤15° (range 5·1–14·9°). Seasonal variations in the correlation between vitamin D intake and biomarker have been reported^(^^32^^)^.

Correlation coefficients were substantially improved when the intake of supplemental vitamin C, folate, and vitamins B_6_ and B_12_ was included, which is in line with a previous validation study for a FFQ using biomarkers as a reference method in Spain^(^^31^^)^. These authors demonstrated that correlation coefficients improved for folate (from *r* 0·12 to 0·53), vitamin C (from *r* 0·18 to 0·20) and vitamin B_12_ (from *r* 0·08 to 0·12) when supplement intake was included. A significant impact of supplement use on plasma vitamin concentrations has also been reported^(^^32^^)^. Our results, together with the above, support the need for nutritional assessment using a FFQ during pregnancy to include assessment of supplement use to achieve valid vitamin estimates.

The present study has several limitations. First, FFQ1 and FFQ2 collected information for the 2 months prior to their administration. Participant recall bias could possibly result in underestimation. A previous validation study using the FFQ asked about food consumption over the previous year and also collected 28- or 14-d dietary records during that year. Although they collected food consumption data over a longer period than in the present study, they found a moderate to good correlation^(^^36^^)^. Furthermore, they evaluated the reproducibility of the FFQ by comparing repeated FFQ at 1-year intervals and demonstrated moderate to high reproducibility for most nutrients^(^^37^^)^. In light of these findings, we consider that participant recall bias for food consumption was likely minimal. Second, this study was conducted at a single perinatal centre in an urban area that accepted pregnant women with complications. Additionally, the mean maternal age was higher than that of the general population in Japan (35·2 *v*. 30 years)^(^^38^^)^ and the participants’ socio-economic status was higher than that of the general population^(^^38^^)^. Indeed, the high educational status may have contributed to the high internal validity of the estimates of nutrients by FFQ. Third, we were unable to investigate the effect of seasons on the validity of the FFQ, because 89·5 % of participants were recruited between May and October 2011, when the average monthly temperature was ≥15° in Tokyo. However, we administered the FFQ twice during each participant's pregnancy. Thus, most of the FFQ2 were administered during the cooler months. Although the effects of plasma volume increase and change in appetite would affect the validity of FFQ2, the results of both FFQ would have been affected by seasonality. Fourth, while although we included the effect of NVP and supplement use during pregnancy, there may have been other unmeasured confounders such as participants’ genotypes for vitamin metabolism^(^^39^^)^ and duration of daily sunlight exposure. Although we investigated the effect of NVP on vitamin intake by stratifying for NVP status, there is still a possibility of residual confounding. Indeed, women with NVP were more likely to be in the lowest educational level. Finally, we used non-fasting blood samples, which may have affected the results because of circadian variation.

In conclusion, our study demonstrated that, in spite of the impact of NVP on dietary intake and serum vitamin concentrations, FFQ is useful for estimating the intake of several important vitamins in early and late pregnancy. Notably, our results highlight the importance of considering NVP when assessing nutritional intake during pregnancy. Given that there is growing interest in maternal nutrition during pregnancy, future epidemiological studies investigating the effect of vitamin intake during pregnancy on various outcomes using the FFQ in Japan are warranted.
